# External validation of the SWEDEHEART score for predicting in-hospital major bleeding among East Asian patients with acute myocardial infarction

**DOI:** 10.3389/fcvm.2022.1001261

**Published:** 2023-01-11

**Authors:** Yabin Liu, Fei Lv, Qucheng Wei, Qiyue Gao, Jun Jiang

**Affiliations:** The Second Affiliated Hospital, Zhejiang University, Hangzhou, Zhejiang, China

**Keywords:** SWEDEHEART score, in-hospital major bleeding, AMI, East Asian, diabetes

## Abstract

**Background:**

Risk scores for predicting in-hospital major bleeding in patients with acute myocardial infarction (AMI) are rare. The Swedish web-system for the enhancement and development of evidence-based care in heart disease evaluated according to recommended therapies (SWEDEHEART) score (SS), consisting of five common clinical variables, is a novel model for predicting in-hospital major bleeding. External validation of SS has not yet been completed.

**Methods and results:**

A retrospective study recruiting consecutive East Asian patients diagnosed with AMI was conducted in the Second Affiliated Hospital, Zhejiang University. The primary endpoint was the ability of SS to predict in-hospital major bleeding, which was defined as Bleeding Academic Research Consortium (BARC) type 3 or 5 bleeding. To validate SS, the discrimination and calibration were assessed in the overall population and several subgroups. The receiver operating characteristic (ROC) curves and the areas under ROC curves (AUCs) were calculated for discrimination. The calibration of SS was evaluated with the unreliability *U* test. A total of 2,841 patients diagnosed with AMI during hospitalization were included, and 1.94% (55) of them experienced in-hospital major bleeding events. The AUC of SS for the whole population was only 0.60 [95% confidence interval (CI), 0.52–0.67], without an acceptable calibration (*p* = 0.001). Meanwhile, the highest AUC (0.72; 95% CI, 0.61–0.82) of SS for the primary endpoint was found in the diabetes subgroup, with an acceptable calibration (*p* = 0.87).

**Conclusion:**

This external validation study showed that SS failed to exhibit sufficient accuracy in predicting in-hospital major bleeding among East Asian patients with AMI despite demonstrating acceptable performance in the diabetic subgroup of patients. Studies to uncover optimal prediction tools for in-hospital major bleeding risk in AMI are urgently warranted.

## 1. Introduction

Although the ischemic risk of acute coronary syndrome (ACS) has been considerably reduced with the development of percutaneous coronary intervention (PCI) and antithrombotic therapy over the last decade, bleeding has always been a common and non-neglectable complication associated with poor prognosis (1), especially in-hospital bleeding under the administration of intensive antiplatelet and anticoagulant medications (2–4). Identifying specific populations at high risk for bleeding is particularly important during daily clinical practice, and current guidelines recommend using the Can Rapid Risk Stratification of Unstable Angina Patients Suppress Adverse Outcomes with Early Implementation of the American College of Cardiology/American Heart Association Guidelines (CRUSADE) score as an acceptable model to deal with this issue (5, 6). However, the CRUSADE score is not suitable for the application among conservatively treated patients and is substantially limited due to the widespread use of radial access during PCI (6). Novel risk models for predicting in-hospital bleeding are rare.

The Swedish web-system for the enhancement and development of evidence-based care in heart disease evaluated according to recommended therapies (SWEDEHEART) score (SS), generated from the large-scale SWEDEHEART registry, is a novel and convenient model including only five clinical variables to predict in-hospital major bleeding following acute myocardial infarction (AMI) (7). Internal–external cross-validation showed that SS had better performance than the CRUSADE score in assessing the registry-defined in-hospital major bleeding risk (*C*-index, 0.80 vs. 0.72) among patients irrespective of a history of PCI (7). Both its favorable discriminative ability and good convenience have qualified SS to be a promising prediction model. However, the derivation cohort of SS included AMI patients from Sweden, and no external validation with a different population has been conducted to further confirm the performance of this model. East Asian patients with AMI, who are considered to have a relatively increased risk of bleeding, should be paid more attention to risk assessment (8, 9). Therefore, we aimed to externally validate SS for predicting in-hospital major bleeding in East Asian AMI patients in order to examine its transportability.

## 2. Materials and methods

### 2.1. Patient population

This was a retrospective cohort study that enrolled consecutive patients diagnosed with AMI in the Department of Cardiology at the Second Affiliated Hospital, Zhejiang University, Hangzhou, China, between June 2018 and June 2021 irrespective of a history of PCI. Major exclusion criteria included being transferred to undergo coronary artery bypass graft surgery during hospitalization and refusing to participate in the study. AMI was defined according to the third universal concept of infarction (10). The demographic data, medical history, laboratory tests, and in-hospital complications (including bleeding events) were strictly recorded in a computer database. The strategy of PCI procedures and periprocedural antithrombotic medications was chosen at physicians’ discretion according to standard guidelines and also recorded. This retrospective study was performed in accordance with the Declaration of Helsinki and was approved by the Ethics Committee of the Second Affiliated Hospital, Zhejiang University (reference no. EC-20220412-1020). All patients involved in this study provided written informed consent.

### 2.2. Endpoints

The primary study endpoint was the predictive accuracy of SS for in-hospital major bleeding in AMI patients. Bleeding events that occurred after PCI during hospitalization were judged by a pair of independent cardiologists. Localization of bleeds and changes in hemoglobin (Hb) values were collected from the hospital information system. In-hospital major bleeding was defined as Bleeding Academic Research Consortium (BARC) type 3 or 5 bleeding, which is the most prevalently used bleeding criterion (11).

### 2.3. Statistical methods

Continuous data are presented as mean ± standard deviation values and were compared using Student’s *t* test if normally distributed or as median and interquartile range (IQR) values and compared by the Wilcoxon rank-sum test if non-parametric. Categorical data are presented as counts and percentages and were compared using Fisher’s exact test.

SWEDEHEART score includes the following five variables: sex, Hb, age, C-reaction protein (CRP) level, and serum creatinine concentration. For patients missing CRP information, a modified SS was also generated (7). For SS calculations, the estimated major bleeding probability was computed as 1/[1 + exp(−bX)], where “bX” varied according to whether CRP data were available or missing (7).

To validate SS, its discrimination was assessed in both the overall and the subgroup datasets. Subgroups included those established according to age (<65 or ≥65 years), the presence or absence of diabetes mellitus (DM), sex, the presence or absence of heart failure (HF), a history of PCI, and the occurrence of ST-segment elevation MI (STEMI) or non-STEMI (NSTEMI). By taking the predicted probability of major bleeding as the test variable and the observed proportion of major bleeding as the state variable, the receiver operating characteristic (ROC) curve was drawn, and the area under the ROC curve (AUC) was calculated (12, 13). Similar to the discrimination, the calibration was also assessed in both the overall and the subgroup datasets. The calibration curve of SS for major bleeding according to the predicted probabilities and the observed proportions was drawn (14), and the calibration of SS was evaluated with an unreliability test (15). A decision curve was plotted (16), and decision curve analysis was performed to judge the clinical use of SS by quantifying the net benefits with different probability thresholds in the overall dataset (17, 18). All *p* values were two-tailed, and *p* < 0.05 was considered to be statistically significant. All statistical analyses were conducted using Stata/SE version 15.0 (StataCorp LLC, College Station, TX, USA) and R version 3.3.1 (R Foundation for Statistical Computing, Vienna, Austria).

## 3. Results

### 3.1. Baseline characteristics

A total of 2,841 patients diagnosed with AMI during hospitalization were included in this retrospective study, of whom 2,324 patients (81.8%) received PCI during hospitalization. In-hospital major bleeding occurred in 55 patients (1.94%) (a flowchart is shown in Supplementary Figure 1). All bleeding events were BARC type 3 bleeding; no case of BARC type 5 fatal bleeding was found in this cohort.

The baseline characteristics of patients with and without in-hospital major bleeds are presented in Table 1. The proportion of a previous MI history in the major bleeding group was significantly greater than that in the no major bleeding group (18.2% vs. 9.4%, *p* = 0.04). Patients in the major bleeding group more often presented with signs or symptoms of HF (30.9% vs. 9.3%, *p* < 0.001) and atrial fibrillation (7.3% vs. 2.1%, *p* = 0.03) at admission. During hospitalization, complications such as shock, new-onset atrial fibrillation, cardiopulmonary resuscitation, and death also occurred more frequently in the major bleeding group (*p* < 0.05 for all). Patients in the major bleeding group had a significantly lower Hb concentration (129 ± 20 g/L vs. 135 ± 20 g/L, *p* = 0.02) and a significantly higher CRP level [median (IQR), 11.2 (5.4–45.1) mg/L vs. 4.2 (2.0–12.8) mg/L; *p* < 0.001] compared to those in the no major bleeding group.

**TABLE 1:** Baseline characteristics.

Characteristic	Total (*n* = 2,841)	Major bleeding (*n* = 55)	No major bleeding (*n* = 2,786)	*p*
Demography				
Age (years)	63.5 ± 12.6	66.7 ± 14.7	63.4 ± 12.6	0.06
Female (sex)	667 (23.5%)	16 (29.1%)	651 (23.4%)	0.34
BMI (kg/m^2^)	24.3 ± 3.2	23.8 ± 3.1	24.3 ± 3.2	0.27
Medical history				
Hypertension	1,713 (60.3%)	35 (63.6%)	1,678 (60.2%)	0.68
Diabetes mellitus	809 (28.5%)	14 (25.5%)	795 (28.5%)	0.76
Previous MI	273 (9.6%)	10 (18.2%)	263 (9.4%)	0.04
Previous PCI	459 (16.2%)	13 (23.6%)	446 (16.0%)	0.14
Previous CABG	9 (0.3%)	1 (1.8%)	8 (0.3%)	0.16
Previous PAD	66 (2.3%)	0 (0.0%)	66 (2.4%)	0.64
Previous stroke	223 (7.8%)	4 (7.3%)	219 (7.9%)	1.00
Chronic heart failure	138 (4.9%)	3 (5.5%)	135 (4.8%)	0.75
Previous bleeding	33 (1.2%)	2 (3.6%)	31 (1.1%)	0.13
Cancer	75 (2.6%)	4 (7.3%)	71 (2.5%)	0.06
Medication at admission				
Proton pump inhibitor	1,653 (58.2%)	37 (67.3%)	1,616 (58.0%)	0.21
β-blocker	2,041 (71.8%)	40 (72.7%)	2,001 (71.8%)	1.00
ACEI/ARB	2,068 (72.8%)	37 (67.3%)	2,031 (72.9%)	0.36
Calcium channel blocker	482 (17.0%)	7 (12.7%)	475 (17.0%)	0.47
Aspirin	2,798 (98.5%)	53 (96.4%)	2,745 (98.5%)	0.20
Clopidogrel	1,886 (66.4%)	37 (67.3%)	1,849 (66.4%)	0.51
Ticagrelor	980 (34.5%)	17 (30.9%)	863 (31.0%)	0.49
Oral anticoagulant	73 (2.6%)	4 (7.3%)	69 (2.5%)	0.051
Presentation				
CPR before admission	70 (2.5%)	3 (5.5%)	67 (2.4%)	0.15
Atrial fibrillation	62 (2.2%)	4 (7.3%)	58 (2.1%)	0.03
Anemia	239 (8.4%)	8 (14.5%)	231 (8.3%)	0.13
Symptoms or signs of HF	276 (9.7%)	17 (30.9%)	259 (9.3%)	<0.001
Shock	23 (0.8%)	2 (3.6%)	21 (0.8%)	0.07
ST elevation	981 (34.5%)	26 (47.3%)	955 (34.3%)	0.06
Laboratory data on admission				
Hemoglobin (g/L)	135 ± 20	129 ± 20	135 ± 20	0.02
Creatinine (mmol/L)	75 (62, 93)	78 (58, 102)	74 (62, 92)	0.74
CRP (mg/L)*	4.3 (2.0, 13.1)	11.2 (5.4, 45.1)	4.2 (2.0, 12.8)	<0.001
Treatment				
Angiography	508 (17.9%)	10 (18.2%)	498 (17.9%)	1.00
PCI	2,324 (81.8%)	45 (81.8%)	2,279 (81.8%)	1.00
Thrombolysis	56 (2.0%)	1 (1.8%)	55 (2.0%)	1.00
GP IIb/IIIa	520 (18.3%)	7 (12.7%)	513 (18.4%)	0.38
Heparin	1,390 (48.9%)	26 (47.3%)	1,364 (49.0%)	0.89
Bivalirudin	546 (19.2%)	9 (16.4%)	518 (18.6%)	0.42
LMWH	876 (30.8%)	15 (27.3%)	861 (30.9%)	0.66
Fondaparinux	413 (14.5%)	10 (18.2%)	403 (14.5%)	0.44
LVEF < 50%	540 (19.0%)	16 (29.1%)	524 (18.8%)	0.08
Complications				
Reinfarction	49 (1.7%)	3 (5.5%)	46 (1.7%)	0.07
In-hospital shock	69 (2.4%)	6 (10.9%)	63 (2.3%)	<0.01
New-onset AF	34 (1.2%)	4 (7.3%)	30 (1.1%)	<0.01
CPR	48 (1.7%)	7 (12.7%)	41 (1.5%)	<0.001
Death	27 (1.0%)	7 (12.7%)	20 (0.7%)	<0.001

Continuous variables are presented using n (%), mean ± standard deviation, or median [interquartile range (IQR)] values. Comparisons between major bleeding and non-major bleeding were made by Student’s t tests or Wilcoxon rank-sum tests as appropriate for continuous variables and Fisher exact tests for categorical variables.

*CRP data were missing for 1,084 patients.

ACEI, angiotensin-converting enzyme inhibitor; AF, atrial fibrillation; ARB, angiotensin receptor blocker; BMI, body mass index; CABG, coronary artery bypass graft; CPR, cardiopulmonary resuscitation; CRP, C-reactive protein; GP, glycoprotein; HF, heart failure; LMWH, low-molecular-weight heparin; LVEF, left ventricular ejection fraction; MI, myocardial infarction; PAD, peripheral artery disease; PCI, percutaneous coronary intervention.

### 3.2. Discrimination of SS

**FIGURE 1:**
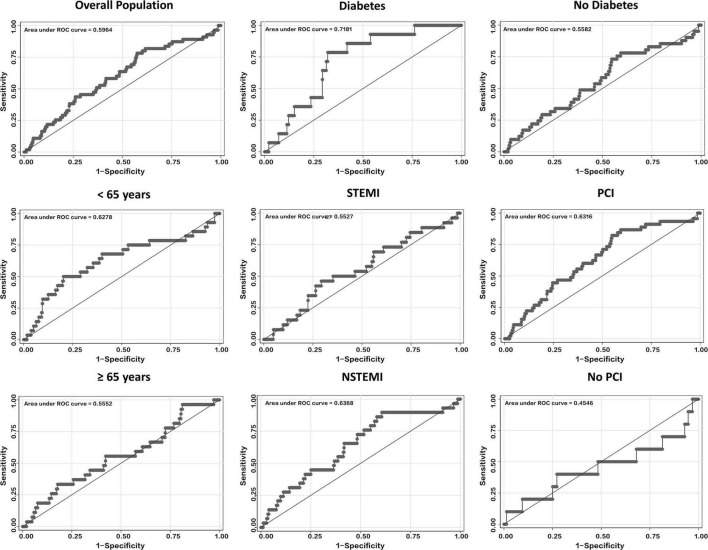
ROCs curves of SS for predicting in-hospital major bleeding in the overall population and several subgroups, including those of age, diabetes, MI type, and PCI. NSTEMI, non-ST-segment elevation myocardial infarction; PCI, percutaneous coronary intervention; ROC, receiver operating characteristics; SS, SWEDEHEART score; STEMI, ST-segment elevation myocardial infarction.

Figure 1 presents the ROCs of SS for in-hospital major bleeding in the whole population and each subgroup. The AUCs and relevant 95% confidence interval (CI) values are presented in Table 2.

**TABLE 2:** AUCs of SS among the overall population and subgroups.

	No.	Bleeding	AUC *C*-index (95% CI)
Overall	2,841	55	0.60 (0.52–0.67)
Age (years)			
<65	1,481	28	0.63 (0.50–0.75)
≥65	1,360	27	0.56 (0.44–0.67)
Diabetes			
No	2,032	41	0.56 (0.47–0.65)
Yes	809	14	0.72 (0.61–0.82)
Sex			
Male	2,174	39	0.59 (0.49–0.68)
Female	667	16	0.59 (0.48–0.71)
Symptoms or signs of HF			
No	2,565	38	0.60 (0.51–0.69)
Yes	276	17	0.51 (0.36–0.66)
PCI			
No	517	10	0.45 (0.22–0.68)
Yes	2,324	45	0.63 (0.55–0.71)
MI type			
NSTEMI	1,860	29	0.64 (0.53–0.74)
STEMI	981	26	0.55 (0.44–0.67)

AUC, area under the receiver operating characteristic curve; CI, confidence interval; HF, heart failure; MI, myocardial infarction; NSTEMI, non-ST-segment elevation myocardial infarction; PCI, percutaneous coronary intervention; SS, SWEDEHEART score; STEMI, ST-segment elevation myocardial infarction.

The AUC of SS for in-hospital major bleeding in the whole population was 0.60 (95% CI, 0.52–0.67) (Figure 1 and Table 2). After subgroup analysis, however, the highest AUC of the score for the primary endpoint, 0.72 (95% CI, 0.61–0.82) (Figure 1 and Table 2), was found in the DM subgroup. Meanwhile, in the non-DM subgroup, the AUC of SS was 0.56 (95% CI, 0.47–0.65) (Figure 1 and Table 2). Across the three subgroups of age < 65 years, a history of PCI, and NSTEMI subgroups, the AUCs ranged from 0.63 to 0.64 (Figure 1 and Table 2). Conversely, the AUCs for the age ≥ 65 years, no history of PCI, and STEMI subgroups were all <0.60, with the lowest AUC of SS for in-hospital major bleeding found in the no history of PCI subgroup (0.45; 95% CI, 0.22–0.68) (Figure 1 and Table 2). The AUCs were all <0.60 in subgroups stratified by sex and HF or not (Table 2 and Supplementary Figure 2).

### 3.3. Calibration of SS

**FIGURE 2:**
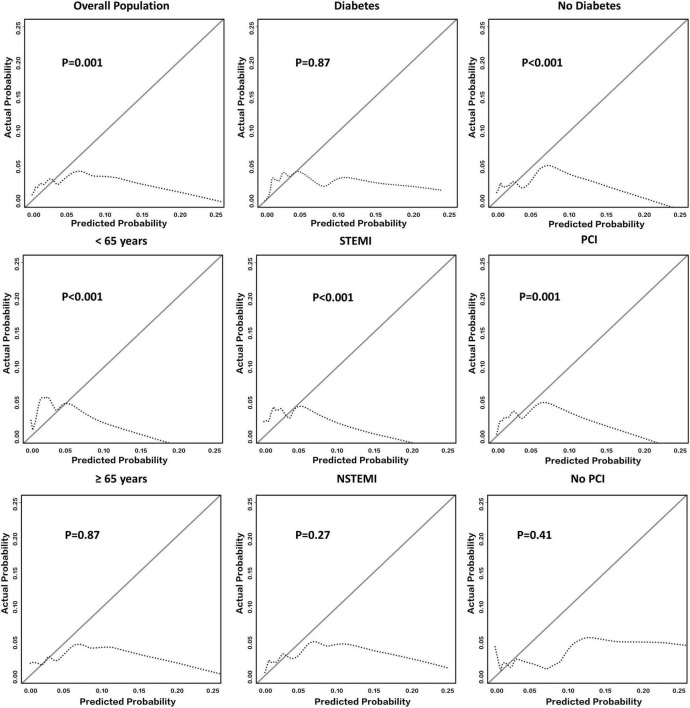
Calibration of SS in all East Asian patients with AMI and their subgroups. It shows the calibration plot of SS for predicting in-hospital major bleeding among the whole East Asian population with AMI and several representative subgroups. Deviations from ideal calibration represent bias in the predicted probability. The figure indicates that the calibration of SS in the overall population was not acceptable (*p* = 0.001). Note that *p* > 0.05 represents an acceptable calibration ability among a single subgroup. AMI, acute myocardial infarction; NSTEMI, non-ST-segment elevation myocardial infarction; PCI, percutaneous coronary intervention; SS, SWEDEHEART score; STEMI, ST-segment elevation myocardial infarction.

The calibration curves of SS for in-hospital major bleeding in the whole cohort (Figure 2) and subgroups (Figure 2 and Supplementary Figure 3) are shown. For the whole population, SS did not reach a level of acceptable calibration for in-hospital major bleeding (*p* = 0.001) (Figure 2). An unacceptable degree of calibration was also found in the male sex, STEMI, non-DM, a history of PCI, and HF diagnosis subgroups, indicating that the observed probabilities were insufficient and the estimated probabilities were low (*p* < 0.05 for all) (Figure 2 and Supplementary Figure 3). However, we confirmed an agreement between observed and predicted in-hospital major bleeding rates in the DM subgroup (*p* = 0.87) (Figure 2). The NSTEMI, no history of PCI, no HF diagnosis, and female sex subgroups also showed an acceptable calibration of SS for the primary endpoint (*p* > 0.05 for all) (Figure 2 and Supplementary Figure 3).

After evaluating discrimination in combination with calibration, SS did not display an eligible level of performance for predicting the risk of in-hospital major bleeding in the whole population. However, among diabetic patients, both the discrimination and calibration of SS were acceptable for the primary endpoint. Results from the NSTEMI subgroup were also acceptable, but the performance of SS was not acceptable among the other subgroups.

### 3.4. Clinical utility of SS

**FIGURE 3:**
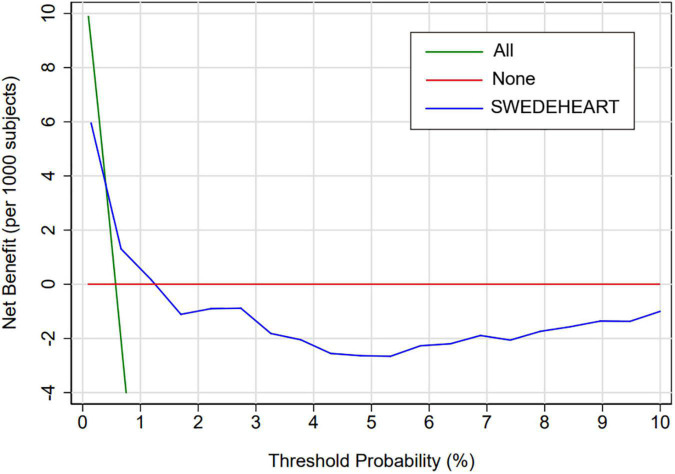
Decision curve analysis of SS among the overall population. Decision curve analysis for SS. The *y*-axis measures the net benefit. The *x*-axis measures the threshold probability. The blue line represents SS. The green line represents the assumption that all patients have an absolute risk of bleeding. The red line represents the assumption that no patients have any risk of bleeding. The decision curve shows that, if the threshold probability of a patient ranges from 0.4 to 1.3%, the patient would benefit from using SS to predict the in-hospital major bleeding risk. SS, SWEDEHEART score.

Figure 3 presents the decision curve of SS for the primary endpoint, which showed that, if the threshold probability of a patient ranges from 0.4 to 1.3%, the patient would benefit from using SS to predict the in-hospital major bleeding risk (Figure 3). After analysis, 1,085 patients had a threshold probability ranging from 0.4 to 1.3%.

## 4. Discussion

This retrospective cohort study first externally validated the predictive accuracy of SS, a novel bleeding risk model, for in-hospital major bleeding among East Asian patients with AMI. This study found that SS did not have good accuracy in predicting the in-hospital major bleeding risk among the whole population. SS had an acceptable performance of discrimination and calibration only among diabetic patients with AMI.

Bleeding events, as the most important adverse events associated with antithrombotic therapy and revascularization strategies, are associated with a relatively higher mortality rate in the management of ACS (19, 20). A recently published cohort study from the large-scale SWEDEHEART registry also determined that upper gastrointestinal bleeding was common and independently associated with all-cause death in AMI patients. The balance of ischemic and bleeding risks for patients with ACS is permanently pursued by contemporary cardiologists. Several recent studies have reported that the rate of in-hospital bleeding remains 3–6% in ACS patients, while the present study found that the rate of in-hospital BARC-defined major bleeding was 1.94% (21, 22). Our study and previous studies collectively prove that bleeding events during hospitalization remain worthy of attention. However, risk scores predicting in-hospital major bleeding are still rare; an existing option, the CRUSADE score, due to its intrinsic deficiency, is now recommended only for patients with angiography by current guidelines (6).

SWEDEHEART score was derived and internally validated from a large-scale real-world SWEDEHEART registry including Caucasian patients and is a new model for predicting the risk of in-hospital major bleeding. In a derivation study, SS showed a better predictive accuracy for in-hospital major bleeding compared to the CRUSADE score among European patients with AMI (*C*-index, 0.80 vs. 0.72) (7). Moreover, SS includes only five common clinical parameters (sex, Hb concentration, age, CRP level, and serum creatinine concentration), making it more convenient than the CRUSADE score (which includes eight parameters). Importantly, the derivation study of SS did not conduct external validation, especially for populations other than Caucasian individuals. Moreover, a judgment of the primary endpoint, in-hospital bleeding, by the study authors was done in accordance with their criterion (readmission due to bleeding), rendering the usefulness of this risk score under universal standardized criteria of bleeding, such as the BARC definition, questionable (11). We, therefore, conducted this retrospective study to externally validate the predictive ability of SS for in-hospital BARC-defined major bleeding (type 3 or 5 bleeding) among East Asian patients with AMI from our site. The East Asian population, which is different from the Caucasian population, is regarded to have a relatively lower ischemic risk but a higher risk of bleeding (referred to as the “East Asian paradox”), and this group deserves to have more attention paid to the bleeding risk (8, 9). However, our results showed that the AUC of SS was only 0.60, and the calibration was also poor, with a great discrepancy between the observed and predicted probabilities (*p* = 0.001). This outcome failed to identify SS as an acceptable risk score for predicting the risk of major bleeding during hospitalization of East Asian patients.

A subgroup analysis among diabetic patients in our study revealed that SS had acceptable discrimination in predicting in-hospital major bleeding, with an AUC value of 0.72, while also presenting an eligible calibration. However, the predictive ability of SS in diabetic AMI patients was also limited. DM is known to be a risk factor not only for recurrent ischemic events but also bleeding among patients with ACS (11, 23, 24). The CRUSADE score may be considered as a tool for predicting in-hospital bleeding events among ACS patients (5). Therefore, an incremental effect of DM on the predictive accuracy of SS from our findings is theoretically reasonable. Although not suitable for the whole population, SS showed an acceptable predictive ability among diabetic East Asian patients in the present external validation study. Further exploration of SS for evaluating the in-hospital major bleeding risk among AMI patients with DM is needed. In total, this external validation study demonstrated that SS may be an alternative method for evaluating the in-hospital major bleeding risk of AMI patients with DM. Meanwhile, the unacceptable predictive ability of SS in other sub-populations supports the need to uncover optimally pragmatic tools for predicting the bleeding risk.

There are several limitations to this study. First, although it met the minimal requirements according to the TRIPOD guideline (25), the sample size of recruited patients was still too small. A large-scale, real-world study focusing on the East Asian population deserves to be conducted. Second, we did not compare the predictive accuracy between SS and the CRUSADE score due to insufficient data availability, but in the earlier derivation study, this comparison was performed (5). Finally, an incremental exploration of the effects of DM on SS was not performed in our study and requires further investigation.

This retrospective external validation study demonstrated that SS does not perform effectively in predicting in-hospital major bleeding among East Asian patients with AMI. However, an acceptable predictive ability of SS was found in AMI patients with DM. Large-scale studies are needed to further validate the accuracy of SS.

## Data availability statement

The raw data supporting the conclusions of this article will be made available by the authors, without undue reservation.

## Ethics statement

Written informed consent was obtained from the individual(s) for the publication of any potentially identifiable images or data included in this article.

## Author contributions

YL was mainly in charge of conducting this observational study. FL was responsible for writing the manuscript. QW contributed to recruiting subjects during the study period. QG was responsible for statistical analysis. JJ led the study conduct and took on the key role of designing, initiating, and conducting this study. All authors contributed to the article and approved the submitted version.
