# Supplementary material for: External validation of the SWEDEHEART score for predicting in-hospital major bleeding among East Asian patients with acute myocardial infarction
Source: Front Cardiovasc Med. 2023 Jan 11;9:1001261. doi: 10.3389/fcvm.2022.1001261 (PMC9873996; doi:10.3389/fcvm.2022.1001261)
Supplement: Supplementary file 1 [file Data_Sheet_1.docx]

**Appendix**

**Figure S1. Study flowchart**

**
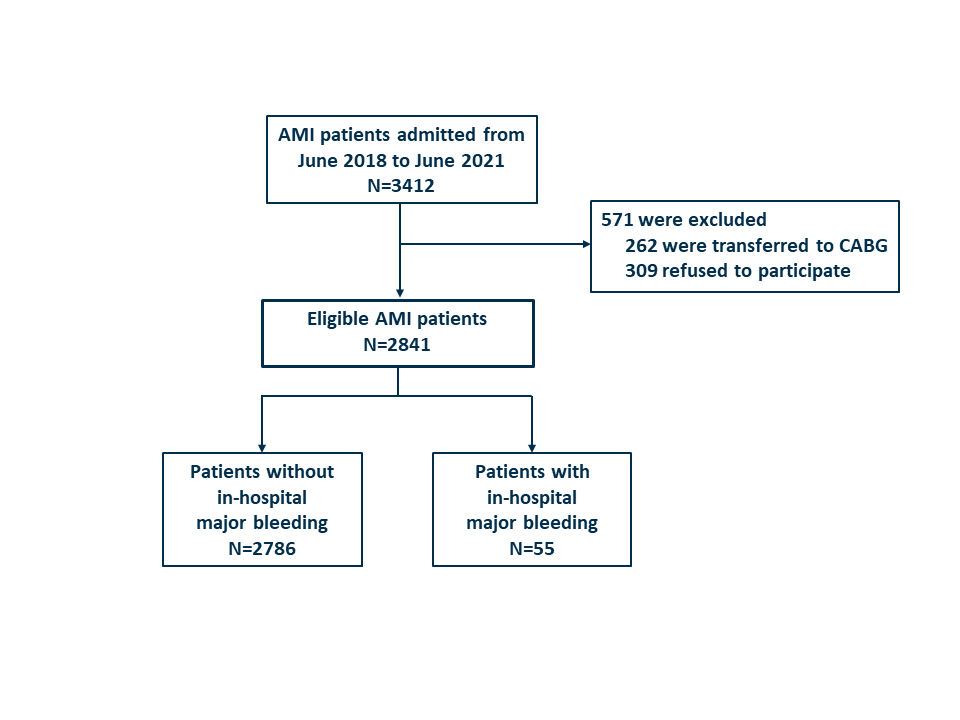
**

Figure S1 shows the flowchart of this external validation study of the SWEDEHEART score. AMI: acute myocardial infarction; CABG: coronary artery bypass grafting.

**Figure S2. Receiver operating characteristics (ROC) curves of SS among subgroups**

**
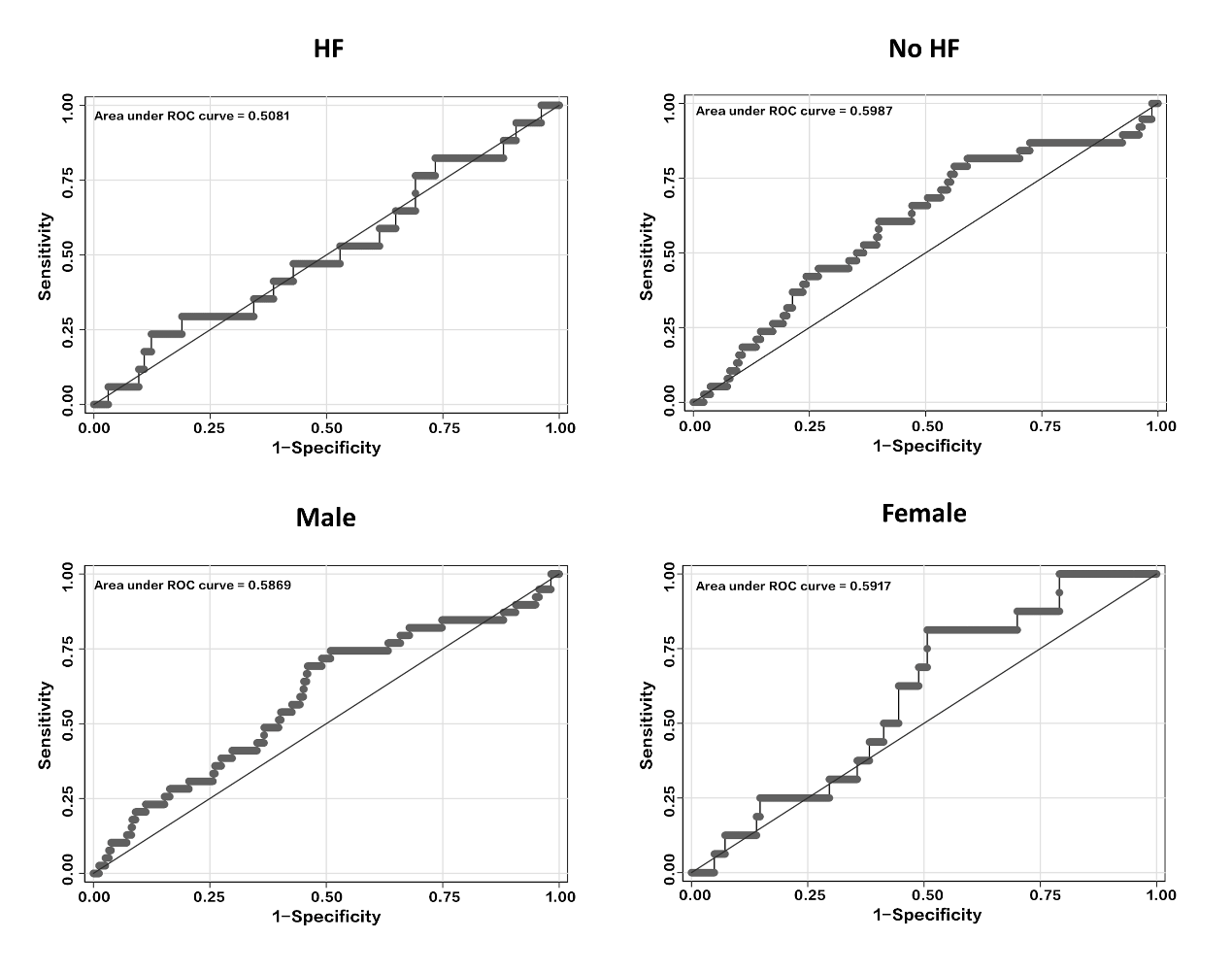
**

Figure S2 shows the ROC curves of SS on predicting in-hospital major bleeding among subgroups including sex and presence or absence of HF. ROC: receiver operating characteristics; SS: SWEDEHEART score; HF: heart failure.

**Figure S3. Calibration of SS among subgroups**

**
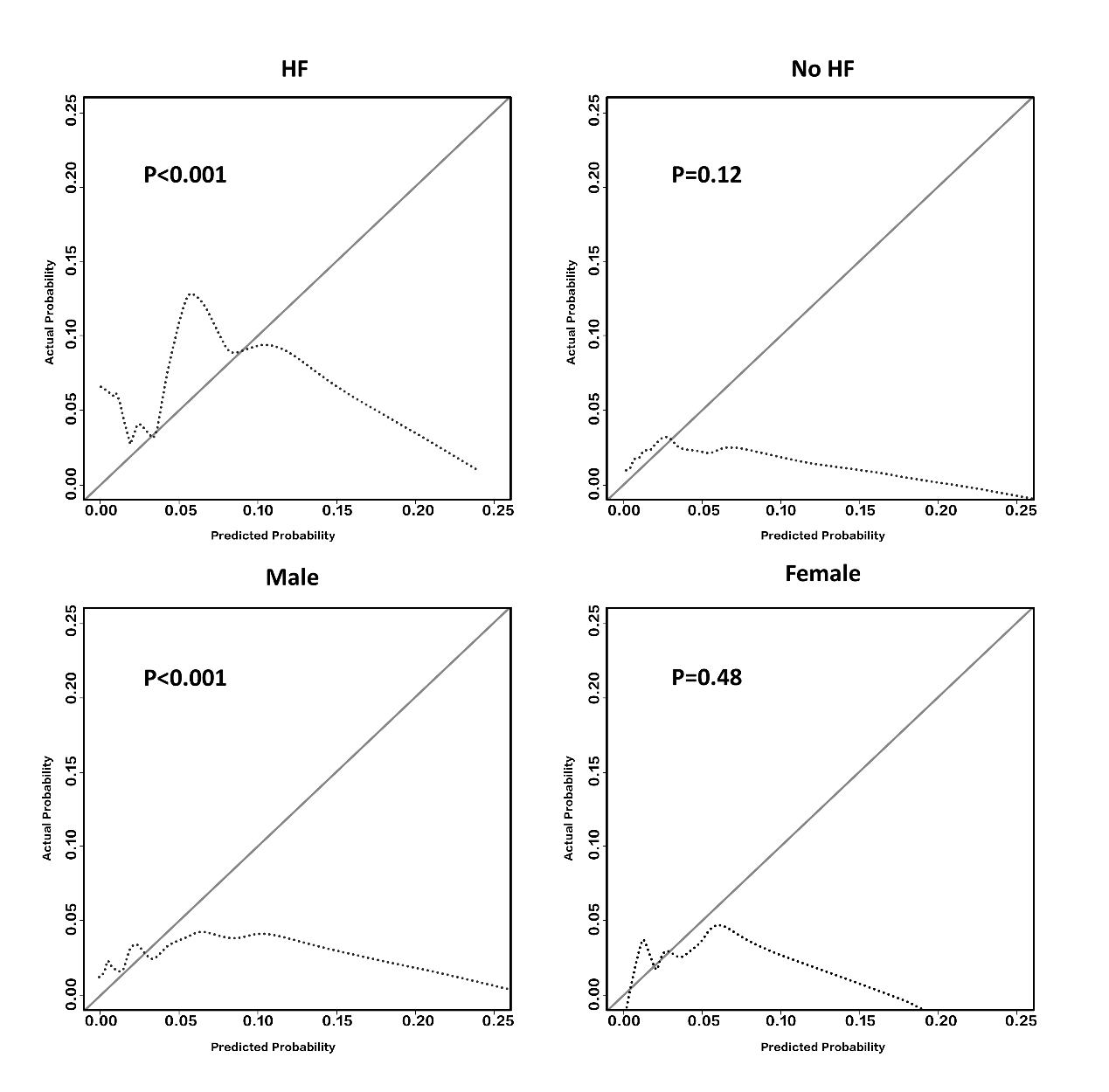
**

Figure S3 shows the calibration plots of SS on predicting in-hospital major bleeding among subgroups including sex and presence or absence of HF. Deviations from ideal calibration represent bias in predicted probabilities. P>0.05 indicates acceptable calibration. SS: SWEDEHEART score; HF: heart failure.
